# Supplementary material for: The safety and efficacy of doxazosin in medical expulsion therapy for distal ureteric calculi: A meta-analysis
Source: PLoS One. 2021 Jan 25;16(1):e0245741. doi: 10.1371/journal.pone.0245741 (PMC7833139; doi:10.1371/journal.pone.0245741)
Supplement: Attachment — Submitted filename: Response to Reviewers.docx [file pone.0245741.s001.docx]

Nov, 2020 15.

RE: manuscript reference number: PONE-D-20-25593.
Title: The safety and efficacy of doxazosin in medical expulsion therapy for distal ureteral calculi: a meta-analysis.

Dear Editors and Reviewers:

Thank you for your comments regarding the submitted paper, which will help us improve it to a better scientific level. We have made detailed revisions in accordance with the reviewer ’s comments. The amendments are highlighted in red in the revised manuscript. Point by point responses to the Reviewers’ comments are listed in the following letter.

I am looking forward to hearing from you.

Sincerely,
Baozhong Yu

Department of Urology

Beijing Chaoyang Hospital, Capital Medical University

8 Gong Ti Nan Road, Chaoyang District, Beijing, 100020, China

Reply to Reviewer：

**Reviewer1**: Thank you for your reviewer’s comments concerning our study by Wang and colleagues. We are very grateful for your valuable comments on the review. The following is my response to each question.
**Question1**: The size of stones should be specified do the authors mean 10 millimeters in diameter or length for example?

Answer1: Thank you very much for Reviewer’s suggestion. We re-modified the summary section and marked the size of the stones in the method section.

**Question2**: The last sentence of the background can be deleted this study does not assess a hypothesis but merely analyzes the results of previously tested hypotheses.

Answer2: Thanks for your suggestion. We also think this sentence is inappropriate. So, we re-edited this sentence.

**Question3**:  I think it would be worthwhile changing the flow of the sections here. the inclusion and exclusion criteria should perhaps precede the literature search. Were there any language restrictions placed on the searches?

Answer3: Thank you very much for Reviewer’s suggestion. We revised the article and put the inclusion and exclusion criteria in front of the literature search. We do not have any language restrictions in the process of searching documents.

**Question4**: although not required by the PRISMA reporting system could the authors tell us how many articles required discussion following discrepancy after independent review?

Answer4: Thank you very much for Reviewer’s suggestion. After we independently reviewed the literature, there are a total of 5 documents that need to be discussed.

The literature was excluded considering that the intervention measures did not meet or the patients were not randomly assigned.

**Question5**: I assume that patient sex rather than gender was extracted?

Answer5: Thank you very much for Reviewer’s suggestion. We noticed that our words were inappropriate. We modified gender to sex.

**Question6**:  Did the authors use a standard method to assess the quality of the study which was the basis of information in figure two?

Answer6: Thank you very much for Reviewer’s suggestion. We evaluated each included study according to the standard of Cochrane's risk assessment tool.

**Question7**: In the sentence following the notes on table one the letters DP&T are incorrectly ordered in terms of explaining the definitions.

Answer7: Thank you for your reminder. In response to the reviewers' comments, we have revised the note and adjusted the order of letters DP&T.

**Question8**: I think the correct description is ureteric rather than ureteral stones. this need correcting throughout

Answer8: Thanks for your suggestion. We noticed that our usage was inappropriate. We checked the article and modified ureteral stone to ureteric stone.

**Question9**: The unit of expulsion time is missing; I assume this is hours but this need stating.

Answer9: Thank you for your reminder. We state that the unit of expulsion time is hours.

**Question10**:  Could the authors give the proportions of exposed patients who experienced each of the listed adverse events?

Answer10: Thanks for your suggestion. In the four studies, there were 212 people in the doxazosin group, of which 7 patients had hypotension, 7 patients had nausea and vomiting, and 5 patients had dizziness.

**Question11**: Are there data for comparative rates of adverse events between doxazosin and tamsulosin?

Answer11: Thank you very much for Reviewer’s suggestion. Four of the eight included studies compared doxazosin and tamsulosin. But only one of the four studies reported adverse drug reactions. Insufficient data are difficult to compare the incidence of drug adverse reaction between doxazosin and tamsulosin.

**Question12**: **The sentence following the paragraph “we found differences in the risk of common adverse reactions…” appears to be redundant. Were the authors able to ascertain whether any medical intervention was required for drug associated adverse events?**

Answer12: Thanks for your suggestion. The sentence following the paragraph “we found differences in the risk of common adverse reactions…” appears to be inappropriate. So, we deleted and revised this sentence. According to the included studies, the symptoms of patients with adverse reactions can generally be relieved. However, it is difficult to determine that medical intervention is not required after adverse events. Therefore, we believed that the sentence in the article is inappropriate, so we deleted this sentence.

**Question13**: Where the authors use the term eviction time, I think they mean expulsion time

Answer13: Thank you for your reminder. We noticed that our words were inappropriate. We modified eviction time to expulsion time.

**Question14**: The authors rightly note that they were unable to include unpublished study data. Did they perhaps investigate whether there were such data? For example, a search of a clinical trials registry might have uncovered other studies of relevance for which there were no results.

Answer14: Thank you very much for Reviewer’s suggestion. Considering that some researchers missed the registration time due to negligence and failed to upload the research and related data to the website. We took this into consideration and said that we they were unable to include unpublished study data. But it is unreasonable to write this without evidence, we deleted this sentence.

**Question15**: In terms of limitations, were included studies English language only? This needs clarification

Answer15: Thanks for your suggestion. We clarified that the research includes not only the English language, but also other languages.

**Reviewer2**: Thank you for your reviewer’s comments concerning our study by Wang and colleagues. We are very grateful for your valuable comments on the review. The following is my response to each question.

**Question1**:  The word "ureteral" is used throughout. Would "ureteric" be a better choice?

Answer1: Thanks for your suggestion. We noticed that our usage was inappropriate. We checked the article and modified ureteral stone to ureteric stone.

**Question2**: There is an inconsistency regarding the episodes of pain between groups, with the doxazocin group experiencing "more episodes of pain" (results, line 7) and "fewer pain episodes" (conclusion). This should be corrected.

Answer2: Thanks for your suggestion. We checked the entire article and noticed inconsistencies in the article, so we revised this part again.

**Question3**: "There are long waiting times for observation and MET". This sentence seems counterintuitive - are the authors suggesting that these treatments take a long time to work, or that there are long wait times to commence treatment, which would be the usual interpretation of this wording. This should be clarified.

Answer3: Thanks for your suggestion. We realize that there is a problem with the wording of this sentence. What we want to clarify is that in addition to conservative treatment and MET, other interventions are high and invasive. We checked and revised this sentence.

**Question4:** "Patient genders" refers to biological sex, not gender. This should be corrected.

Answer4: Thank you very much for Reviewer’s suggestion. We noticed that our words were inappropriate. We modified gender to sex.

**Question5:** The claim that 1 to 2% of hospital admissions are due to ureteral stones should be referenced or removed. "70% of ureteral stones develop within the lower part of the ureter". Is this correct? surely stones form in the kidney and then move to the ureter? Either way it needs referencing.

Answer5: Thank you very much for Reviewer’s suggestion. We noticed that there was a problem with this part of the statement, so we revised this sentence again.

**Question6**: The discussion section is overly long and repeats much of the method and background. It should be heavily edited or rewritten to focus on discussion the findings of the work, rather than reahashing previous sections.

Answer6: Thank you very much for Reviewer’s suggestion. We realized that the discussion section was too long and repeated many methods and backgrounds. So, we re-edited or rewritten the discussion part.

**Question7**: Limitations; the limitation of the study not reporting their SD is a limitation of the statistical method, not of the software. The sentence referring to "Review Manager" should be edited.

Answer7: Thanks for your suggestion, we realize that this sentence is wrong. We checked and revised this sentence.

Sincerely,

Baozhong Yu
