# Supplementary material for: The safety and efficacy of doxazosin in medical expulsion therapy for distal ureteric calculi: A meta-analysis
Source: PLoS One. 2021 Jan 25;16(1):e0245741. doi: 10.1371/journal.pone.0245741 (PMC7833139; doi:10.1371/journal.pone.0245741)
Supplement: Attachment — Submitted filename: Response to Reviewers.docx [file pone.0245741.s002.docx]

Dec, 2020 17.

RE: PONE-D-20-25593R1.
Title: The safety and efficacy of doxazosin in medical expulsion therapy for distal ureteral calculi: a meta-analysis.

Dear Editors and Reviewers:

Thank you for your comments regarding the submitted paper, which will help us improve it to a better scientific level. We have made detailed revisions in accordance with the reviewer ’s comments. Point by point responses to the Reviewers’ comments are listed in the following letter.

I am looking forward to hearing from you.

Sincerely,
Baozhong Yu

Department of Urology

Beijing Chaoyang Hospital, Capital Medical University

8 Gong Ti Nan Road, Chaoyang District, Beijing, 100020, China

**Reply to Reviewer：**

**Reviewer1**: Thank you for your reviewer’s comments concerning our study by Wang and colleagues. We are very grateful for your valuable comments on the review. The following is my response to each question.
**Question1: In the final sentence of the introductory section, the authors might replace the words “which may find “with “and hypothesized that “**

Answer1: Thank you very much for Reviewer’s suggestion. We noticed that our words were inappropriate. We modified “which may find” to “and hypothesized that”.

**Question2: In the second line of the study selection section, the word randomized is repeated in the same sentence. Perhaps the authors might replace the 1st “randomized" with either recruited or enrolled?**

Answer2: Thanks for your suggestion. We noticed that our words were inappropriate. We modified the 1^st^ “randomized” to “recruited”.

**Question3:  In the literature search section, the authors should state that there were no language limitations on their search or retrieval**

Answer3: Thank you very much for Reviewer’s suggestion. We revised the article and declare that there are no language restrictions when searching or retrieving documents.

**Question4:** **In the expulsion time section the authors omit the units of time measured in their text**

Answer4: Thank you very much for Reviewer’s suggestion. In the expulsion time section, the unit of time measured in the text is days. And reflect the time unit in the text

**Question5:** **In the pain episodes section, perhaps “****compared with placebo treatment, doxazosin treatment led to a statistically significant reduction in pain episodes”?**

Answer5: Thank you very much for Reviewer’s suggestion. We noticed that this sentence was inappropriate. As the reviewer said, what we want to express is that the doxazosin group can reduce the frequency of pain episodes compared with the placebo group. We checked and revised this sentence.

**Question6:** **In the drug adverse reaction in section, in the text the authors should specify the comparator where they quote the odds ratio**

Answer6: Thank you very much for Reviewer’s suggestion. We reviewed this part and made it clear that the comparators were the placebo group and the doxazosin group. We checked and revised this part.

**Question7:** **In the expulsion time section when comparing doxazosin to tamsulosin, the authors repeat doxazsin in each group and the unit of time is missing**

Answer7: Thank you for your reminder. In the expulsion time section, the unit of time measured in the text is days. And reflect the time unit in the text.

**Reviewer2**: Thank you for your reviewer’s comments concerning our study by Wang and colleagues. We are very grateful for your valuable comments on the review. The following is my response to each question.

**Question1:  Methods: Analysis. "When the I2 value suggested very low heterogenicity" - the cut off for "very low" is not given and should be added.**

Answer1: Thanks for your suggestion. I^2^ <50% indicates low heterogeneity. When the I^2^ value is less than 50%, the fixed effects model is used. We checked and revised this sentence.

**Question2:** **Discussion: "The mechanism of action of alpha-adrenergic blockers [23] involves the relaxation of the distal ureter by decreasing smooth muscle tension instead of eliminating its activity" this sentence is unclear and should be reworded.**

Answer2: Thanks for your suggestion. We noticed that the sentence was unclear, so we revised it again to make it more concise and clearer.

**Question3:** **The following sentence "In 2005, Sigala et al. [22] determined that the distal ureter expresses higher levels of α1-adrenoceptor mRNA than the proximal and medial ureters."** **doesn't really add much and could be removed.**

Answer3: Thanks for your suggestion. We realized that this sentence doesn't really add much. So, we removed this sentence

**Question4: In the conclusions you state "We concluded that doxazosin has potential as a MET for ureteric stones."** **It seems that your work demonstrates more than this, and the conclusion could be firmer.**

Answer4: Thank you very much for Reviewer’s suggestion. We have revised the conclusion part. Make the conclusion firmer.

Sincerely,

Baozhong Yu
